# Short-Course Induction Treatment with Intrathecal Amphotericin B Lipid Emulsion for HIV Infected Patients with Cryptococcal Meningitis

**DOI:** 10.1155/2015/864271

**Published:** 2015-09-10

**Authors:** Gerardo Alvarez-Uria, Manoranjan Midde, Raghavakalyan Pakam, Pradeep Sukumar Yalla, Praveen Kumar Naik, Raghuprakash Reddy

**Affiliations:** ^1^Department of Infectious Diseases, RDT Bathalapalli Hospital, Kadiri Road, Bathalapalli 515661, India; ^2^Department of Microbiology, RDT Bathalapalli Hospital, Kadiri Road, Bathalapalli 515661, India

## Abstract

Cryptococcal meningitis (CM) is a common cause of death among HIV infected patients in developing countries, especially in sub-Saharan Africa. In this observational HIV cohort study in a resource-limited setting in India, we compared the standard two-week intravenous amphotericin B deoxycholate (AmBd) (Regimen I) with one week of intravenous AmBd along with daily therapeutic lumbar punctures and intrathecal AmB lipid emulsion (Regimen II) during the intensive phase of CM treatment. 78 patients received Regimen I and 45 patients received Regimen II. After adjustment for baseline characteristics (gender, age, altered mental status or seizures at presentation, CD4 cell count, white blood cells, cerebrospinal fluid white cells, and haemoglobin), the use of Regimen II was associated with a significant relative risk reduction in mortality (adjusted hazard ratio 0.4, 95% confidence interval, 0.22–0.76) and 26.7% absolute risk reduction (95% confidence interval, 9.9–43.5) at 12 weeks. The use of Regimen II resulted in lower costs of drugs and hospital admission days. Since the study is observational in nature, we should be cautious about our results. However, the good tolerability of intrathecal administration of AmB lipid emulsion and the clinically important mortality reduction observed with the short-course induction treatment warrant further research, ideally through a randomized clinical trial.

## 1. Introduction

Cryptococcal meningitis (CM) is a common opportunistic infection in HIV infected patients with severe immunosuppression. In middle- and low-income countries, the mortality of CM ranges from 55% to 70% [1, 2]. In sub-Saharan Africa, CM accounts for 10–20% of deaths among HIV infected patients [1]. Globally, it is estimated that approximately 957,900 cases of CM occur each year, resulting in 624,700 deaths [1].

The World Health Organization (WHO) recommends two-week induction treatment with amphotericin B deoxycholate (AmBd) and flucytosine or fluconazole, followed by eight-week consolidation treatment with oral fluconazole [3]. However, a two-week treatment with AmBd is not feasible for many resource-poor settings because it is frequently associated with nephrotoxicity and requires a prolonged admission in a health-care facility with frequent monitoring of the renal function and electrolytes [4]. The nephrotoxicity of AmBd occurs mainly during the second week of therapy, and severe renal toxicity is rare when the treatment is shortened to seven days or less [5–7].

Previous studies have shown strong correlation between the rate of fungal clearance in cerebrospinal fluid (CSF) and survival in CM. AmB has a strong fungicidal activity against* Cryptococcus*, but AmBd and lipid formulations of AmB have poor penetration into CSF [8, 9]. To achieve higher concentration in CSF, intrathecal administration of AmB has been used for the treatment of CM in China, and observational studies suggest that it could be associated with improved survival [10]. However, AmBd has a direct irritant effect, and intrathecal administration of AmBd might be poorly tolerated because of inflammation of the meninges and nerve roots [11, 12]. Previous studies in humans and animals indicate that intrathecal administration of lipid formulations of AmB is better tolerated than AmBd [13–15]. Furthermore, data from a murine model suggest that the combination of intravenous antifungal agents with intrathecal administration of liposomal AmB could be beneficial in terms of survival and reduction of fungal load in CSF [16, 17].

The aim of this study was to compare the standard two-week intravenous therapy with an induction regimen including one week of intravenous AmBd and intrathecal AmB lipid emulsion (AmBle), both regimens accompanied by oral fluconazole for two weeks, in patients from a prospective HIV cohort study in India.

## 2. Methods

### 2.1. Setting

The Vicente Ferrer HIV Cohort Study (VFHCS) is a prospective cohort study of HIV infected patients who have attended the Rural Development Trust Hospital in Bathalapalli, Anantapur District, AP, India. The VFHCS is registered at ClinicalTrials.gov (number NCT02454569). The hospital belongs to a nongovernmental organization and provides medical care to HIV infected people free of charge. In our setting, 72% of the population live in rural areas [18], and the HIV epidemic is largely driven by heterosexual transmission and it is characterized by low CD4 cell counts at presentation, poor socioeconomic conditions, and high levels of illiteracy [19–21].

### 2.2. Definitions and Design

All HIV infected patients from the VFHCS database diagnosed with CM from 1 October 2010 to 31 January 2015 were included. Patients who did not complete four weeks of follow-up since the initiation of CM treatment were excluded. There were no other exclusion criteria. The diagnosis of CM was based on the presence of* Cryptococcus* antigens in CSF by latex agglutination [2]. Fungal burden in CSF was not measured. Altered mental status was defined in patients with confusion, disorientation, coma, or low consciousness.

### 2.3. Treatment

All patients were admitted to the hospital. As in most low- and middle-income countries, flucytosine is not available in India, so fluconazole was used during the induction phase.

Before 15 June 2013, patients were treated with the standard two-week induction treatment with intravenous AmBd 0.7–1 mg/kg and oral fluconazole 1200 mg once daily followed by 600 mg once daily for eight weeks (consolidation phase) and 200 mg once daily thereafter (maintenance phase) [3]. Owing to the lack of disposable manometers, CSF opening pressure was not measured. Therapeutic lumbar punctures with removal of at least 20 mL of CSF (typically 30–40 mL) were performed on alternate days during the first six days of induction therapy, and according to the apparent opening pressure (rapid flow of CSF through the lumbar puncture needle) and patient's symptoms, such as blurred vision, headache, or altered mental status.

The procedure for therapeutic lumbar punctures and the treatment used during the consolidation and the maintenance phases were identical in both groups.

After 15 June 2013, patients received intravenous AmBd 0.7 mg/kg once daily for seven days, intrathecal AmBle 2.5 mg (Amphomul, Bharat Serums and Vaccines, India) once daily for seven days [22, 23], and oral fluconazole 600 mg twice daily for 14 days. Lumbar punctures were performed daily and, after removing at least 20 mL of CSF (typically 30–40 mL), a solution of 0.5 mL (2.5 mg) of AmBle in four mL of 10% dextrose was inoculated slowly into the subarachnoid space. After removing the lumbar puncture needle, patients were asked to lay flat on the bed for one hour. To assess toxicity, patients were asked daily about symptoms related to the local irritating effect of AmB such as lumbar pain, leg pain, vomiting, or retention of urine or stools after the intrathecal administration of AmBle.

AmBle was presented in a vial of 50 mg/10 mL emulsion, so only one vial was used for each patient with CM. The cost of each vial was 1,080 Indian rupees (around 17 USD in March 2015). While the total cost of drugs used in the two-week standard AmB treatment was 3,465 Indian rupees (around 55 USD in March 2015), the total cost of the one-week short-intrathecal AmB treatment was 2,932 Indian rupees (around 47 USD in March 2015).

Patients from both groups received one litre of normal saline solution with 20 mmol of potassium chloride before each infusion of intravenous AmBd [3], and patients not taking antiretroviral therapy (ART) were recommended to initiate ART after four weeks of the initiation of CM treatment. There were no changes in the availability of ART during the two periods of the study.

### 2.4. Statistical Analysis

Statistical analysis was performed using Stata Statistical Software (Stata Corporation, Release 12.1; College Station, Texas, USA). Time-to-event methods were used. Time was measured from treatment initiation to death. Patients who did not die during the first 12 weeks of treatment were censored at week 12 or at their latest visit date, whatever occurred first. Univariate and multivariate analyses were performed with Cox regression proportional hazard models. The proportional hazard assumption was assessed performing log-log survival curves based on Schoenfeld residuals [24]. In a sensitivity analysis, we also performed a multivariate analysis using flexible parametric survival analysis with three degrees of freedom [25]. The selection of variables included in the multivariate analyses was based on the results of a large cohort study investigating determinants of mortality of HIV-associated cryptococcal meningitis and the availability of data in our cohort [26]. The study was approved by the Ethics Committee of the Rural Development Trust Hospital.

## 3. Results

### 3.1. Study Population

During the study period, 126 patients were treated from CM. Two patients who received the standard AmB regimen and one patient who received the short-intrathecal AmB regimen did not complete four weeks of follow-up and were excluded. 123 patients were included in the final analysis, 78 patients in the standard AmB regimen group, and 45 patients in the short-intrathecal AmB regimen group. Baseline characteristics and between group differences are described in Table 1. We did not find significant differences between groups, although the proportion of patients with altered mental status or seizures was higher in the standard AmB regimen group (34.6% versus 22%, *P* value = 0.086). Of those not on ART at the time of starting CM treatment, the median time from CM treatment to initiation of ART was 35 days (interquartile range: 29–51) in the standard AmB regimen group and 36 days (interquartile range: 26–48) in the short-intrathecal AmB regimen group.

### 3.2. Mortality

**Figure 1:**
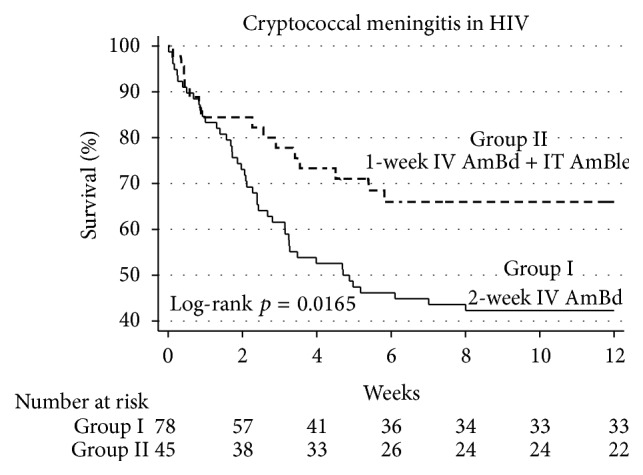
Kaplan-Meier survival estimates by treatment group. IV AmBd, intravenous amphotericin B deoxycholate. IT AmBle, intrathecal amphotericin B lipid emulsion.

**Figure 2:**
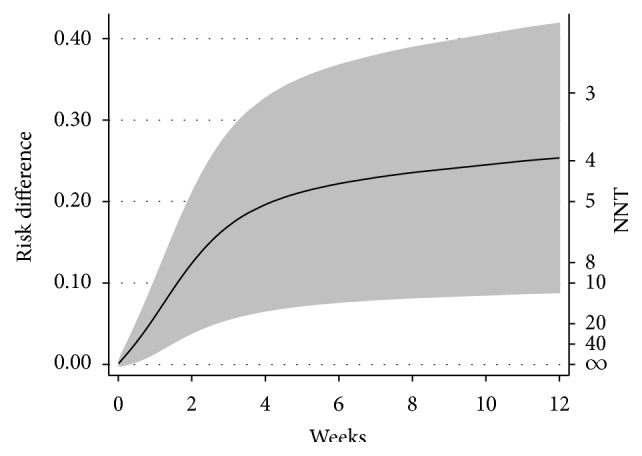
Adjusted risk difference and number needed to treat using flexible parametric survival methods. NNT: number needed to treat.

Sixty patients died during the study period, 45 in standard AmB group, and 15 in the short-intrathecal group. Kaplan-Meier survival estimates by treatment group are shown in Figure 1. Compared with patients who received the standard AmB regimen, patients in the short-intrathecal AmB regimen group had significant lower mortality (*P* = 0.0165). Univariate and multivariate analyses of factors associated with mortality are described in Table 2. Compared with the standard AmB regimen, patients who received the short-intrathecal AmB regimen had a significant lower risk of death in univariate (hazard ratio (HR) 0.5, 95% confidence interval [CI], 0.28–0.89, *P* = 0.019) and multivariate analyses (adjusted HR 0.4, 95% CI, 0.22–0.76, *P* = 0.005). We also performed a multivariate analysis using flexible parametric survival methods, showing similar results (adjusted HR 0.4, 95% CI, 0.21–0.75, *P* = 0.004). Adjusted risk differences and numbers needed to treat are shown in Figure 2. The use of the short-intrathecal AmB regimen was associated with a 26.7% absolute risk reduction (95% CI, 9.9–43.5) in mortality at 12 weeks.

### 3.3. Tolerability of Intrathecal AmBle

Intrathecal administration of AmBle was well tolerated. We did not observe drug adverse reactions described in previous studies using intrathecal AmBd such as lumbar pain, leg pain, vomiting, prostration, or altered mental status [11, 12]. Of 45 patients in the short-intrathecal AmB regimen group, only one patient developed acute urinary retention, which resolved after one day.

## 4. Discussion

In this observational cohort study in a resource-limited setting, the use of a short-course intravenous AmBd regimen along with intrathecal AmBle was well tolerated and was associated with a clinically important reduction in mortality compared with the standard of care in low- and middle-income countries.

The WHO recommends two weeks of intravenous AmBd and flucytosine or fluconazole during the intensive phase of CM treatment. However, this regimen is impractical in resource-poor settings because of the cost of medicines and hospital care [4]. Therefore, fluconazole monotherapy is the treatment most commonly used in low- and middle-income countries, albeit its lower efficacy compared with AmB regimens [4]. The short-intrathecal AmB regimen was associated not only with an increased survival but also with a lower cost in terms of days of hospitalization and drugs. In addition, one week of intravenous AmBd could be given safely without laboratory monitoring, as long as hydration and potassium supplements are provided [7, 27].

Unlike previous experiences with intrathecal AmBd [11, 12], intrathecal AmBle was very well tolerated, and only one patient developed a transient urinary retention. This is in accordance with the case reports of patients receiving other lipid forms of AmB intrathecally and with the results from animal models [13–15, 17]. To our knowledge, this is one of the first studies investigating the effectivity of a lipid form of AmB administered intrathecally in CM. A previous study performed in mice showed that combination therapy including intrathecal liposomal AmB and fluconazole was able to reduce mortality compared with intravenous liposomal AmB monotherapy [16]. Moreover, mice treated with intrathecal liposomal AmB had minimal inflammatory signs in the meninges [16, 17].

In developed countries, disposable manometers are used to control the intracranial pressure [28]. In our study, we did not measure the intracranial pressure of patients with CM. However, this reflects the situation in most resource-limited settings, where disposable manometers are rarely used due to cost, unavailability, and safety issues. A study performed in South Africa and Uganda suggests that therapeutic lumbar punctures can have a dramatic effect on survival of CM regardless of the initial intracranial pressure [29]. Although at least four therapeutic lumbar punctures were performed in the standard AmB regimen group, the mortality reduction seen with the short-intrathecal AmB regimen could be related to the fact that patients in this group undertook more therapeutic lumbar punctures. We speculate that the combination of therapeutic lumbar punctures and intrathecal administration of AmBle might have achieved a more rapid clearance of the fungal load in CSF. However, new studies are needed to confirm these hypotheses.

In some African countries, refusal of lumbar punctures is common due to the belief among patients and relatives that the procedure can cause death [30]. However, in our setting, lumbar puncture refusal was rare, and patients frequently referred to improvement of their symptoms after the procedure. In our experience, the fact that “spinal taps” were used as means of administrating antifungal medication in a “difficult-to-reach” space helped improve the acceptability of therapeutic lumbar punctures among patients and doctors. New studies are needed to evaluate the acceptability of frequent lumbar punctures in settings with higher HIV prevalence in the general population such as sub-Saharan Africa.

The study has some limitations. Given the observational nature of the study, we must be cautious about the survival benefits of the new short-course induction regimen. This was an observational study using routine clinical data in a resource-poor setting. The selection of treatment was not randomized and the two regimens compared in this study were given at different periods of time, so it is possible that our results could be influenced by unknown confounders not included in the multivariate analysis. In addition, we did not measure the CSF opening pressure or surrogates for fungal burden in CSF such as quantitative cultures or* Cryptococcus* antigen titres, which are important predictors of mortality [26]. On the other hand, unlike clinical trials, no patient was excluded because of the severity of the disease, so our results could be generalized to other resource-poor setting where patients are diagnosed with CM at advanced stages [31].

## 5. Conclusions

The study demonstrates that intrathecal administration of AmBle is well tolerated. In addition, the short induction therapy with intravenous AmBd along with daily therapeutic lumbar punctures and intrathecal AmBle achieved a clinically important reduction in mortality compared with the standard of care in low- and middle-income countries. Being an observational study, we must be cautious about the survival benefits of the new regimen. Still, the mortality of CM in resource-poor setting is unacceptably high, and our findings deserve further research.

## Figures and Tables

**Table 1:** Baseline characteristics by treatment group.

	Value	Standard Rx (*n* = 78)	Short i.t. Rx (*n* = 45)	*P* value
Gender	Male	53 (67.95)	35 (77.78)	0.245
	Female	25 (32.05)	10 (22.22)	

Abnormal mental status or seizures	No	51 (65.38)	36 (80)	0.086
	Yes	27 (34.62)	9 (20)	

On antiretroviral therapy	No	39 (50)	20 (44.44)	0.552
	Yes	39 (50)	25 (55.56)	

CD4 cell count (cells/*μ*L)	0–25	20 (25.64)	10 (22.22)	0.753
	26–50	18 (23.08)	12 (26.67)	
	51–100	11 (14.1)	9 (20)	
	>100	29 (37.18)	14 (31.11)	

Age, mean (SD)	Years	36.72 (9.25)	38.29 (9.13)	0.362

White blood cells, mean (SD)	Cells/nL	6.24 (4.74)	6.42 (5.71)	0.862

Hemoglobin, mean (SD)	g/dL	9.18 (2.49)	9.43 (1.9)	0.538

CSF white cells, median (IQR)	Cells/mL	17 (6–76)	16 (4–40)	0.52

CSF: cerebrospinal fluid; IQR: interquartile range; i.t.: intrathecal; Rx: treatment.

Data are presented as number (%) unless otherwise indicated. Standard Rx: two-week intravenous amphotericin B deoxycholate (AmBd). Short i.t. Rx: one week of intravenous AmBd and intrathecal AmB lipid emulsion. *P* values were calculated using Chi^2^ test for categorical variables and *t*-test (not assuming equal variances) for continuous variables.

**Table 2:** Univariate and multivariate analysis of factors associated with mortality using Cox proportional hazard methods.

	Hazard ratio	Adjusted hazard ratio
Female	0.794 (0.442–1.424)	0.800 (0.431–1.484)
AMS or seizures	2.337^*∗*^ (1.394–3.918)	1.716 (0.982–2.998)
CD4 count (cells/mcl)		
0–25	1 (reference)	1 (reference)
26–50	1.115 (0.551–2.255)	0.875 (0.405–1.892)
51–100	0.903 (0.395–2.064)	1.116 (0.476–2.614)
>100	1.020 (0.522–1.993)	0.970 (0.487–1.934)
On ART	0.896 (0.54–1.486)	1.012 (0.588–1.739)
Age (years)	1.015 (0.989–1.043)	1.013 (0.985–1.042)
WBC (cells/nL)	1.097^*∗*^ (1.054–1.143)	1.147^*∗*^ (1.092–1.204)
Haemoglobin (g/dL)	0.922 (0.820–1.036)	0.860^*∗*^ (0.758–0.976)
CSF WC (cells/mL)	0.995^*∗*^ (0.991–0.999)	0.995^*∗*^ (0.992–0.999)
Treatment		
Standard	1 (reference)	1 (reference)
Short i.t. course	0.496^*∗*^ (0.276–0.890)	0.403^*∗*^ (0.215–0.756)

^*∗*^ *P* value < 0.05. AMS: altered mental status; ART: antiretroviral therapy; CSF WC: cerebrospinal fluid white cells; i.t.: intrathecal; Rx: treatment; WBC: white blood cells.
